# Human placental cells are resistant to SARS-CoV-2 infection and replication

**DOI:** 10.12688/wellcomeopenres.20514.1

**Published:** 2024-04-18

**Authors:** Nagisa Yoshida, Jake R. Thomas, Anna Appios, Matthew P. Brember, Irving L.M.H. Aye, James R. Edgar, Andrew E. Firth, Betty Y.W. Chung, Naomi McGovern, Hazel Stewart

**Affiliations:** 1 Department of Pathology, University of Cambridge, Cambridge, England, UK; 2 Centre for Trophoblast Research, University of Cambridge, Cambridge, England, UK; 3 Department of Obstetrics and Gynaecology, University of Cambridge, Cambridge, England, UK

**Keywords:** SARS-CoV-2, trophoblast, placenta

## Abstract

**Background:**

Infection during pregnancy with SARS-CoV-2 can have a serious impact on both maternal and foetal health. Clinical studies have shown that SARS-CoV-2 transmission from the mother to the foetus typically does not occur. However, there is evidence that SARS-CoV-2 can infect the placenta *in utero*. Here we sought to quantify the permissiveness of placental cells to SARS-CoV-2 infection and to determine if they support viral release.

**Methods:**

By using publicly available single-cell RNA sequencing (scRNAseq) data sets and confocal microscopy we compared ACE2 transcript and protein expression across human first trimester and term placental cells. ACE2 transcripts are found in a range of placental cell types across gestation, including trophoblast. However, ACE2 protein expression does not significantly change across placental cell types from first trimester to term.

**Results:**

Using *in vitro* infection assays, we demonstrate that 0.5±0.15 % of term trophoblast cells can be infected with SARS-CoV-2 while primary placental fibroblasts and macrophages, and JEG-3, JAR and HUVEC cell lines are resistant to infection. Furthermore, primary trophoblast cells poorly support viral release while JEG-3 cells allow relatively high levels of viral release.

**Conclusions:**

The low level of viral release by primary placental cells provides insight into how the virus is impaired from crossing the placenta to the foetus.

## Plain Language Summary

The placenta is a fetal-derived organ that starts to form at 5 days post-conception. It forms the interface between the mother and foetus, mediating the transmission of oxygen and nutrients. The placenta also acts an important physical barrier so that only select pathogens can cross to reach the foetus.

Since, the COVID pandemic in 2020 there has been discrepancy in the field over the permissiveness of placental cells to SARS-CoV-2. Infection with SARS-CoV-2 during pregnancy can have a serious impact on both the mother and the unborn child. While newborns infected with SARS-CoV2 have been identified, it remains unclear if infection can occur *in utero*. With this study the researchers evaluated if different placental cell types could be infected by SARS-CoV-2. The findings from this study will help researchers and medical professionals to understand how mother-to-child transmission is impaired *in utero*.

We used a combination of relevant laboratory cell lines and primary samples from study participants (including trophoblast cells). As well as investigating infection levels, we also examined their expression of cellular proteins required for virus cell entry.

The study found that these proteins are expressed at low levels in a range of placental cell types. However, their expression levels do not significantly change during pregnancy. About 0.5% of full-term trophoblast cells can be infected with SARS-CoV-2 and these cells release very few infectious virus particles. Other placental cell types are almost entirely resistant to infection.

Together, these factors reduce the ability of this virus to infect the placenta and cross it to the foetus. This study provides further understanding of the rare cases where SARS-CoV-2 can be found in trophoblast cells but does not transmit further.

## Introduction

Infection with severe acute respiratory syndrome coronavirus 2 (SARS-CoV-2) is an increased risk factor for maternal and foetal health during pregnancy. Symptomatic COVID-19 infections are more likely to result in admittance to the intensive care unit (ICU), preterm deliveries and other adverse maternal and neonatal outcomes when compared to non-pregnant infected women ^1– 4^. Vaccination is protective against these outcomes ^5^. Vertical transmission of SARS-CoV-2 to the foetus typically does not occur even when there is evidence of placental infection ^6– 16^.

SARS-CoV-2 is an enveloped positive-sense RNA virus that relies on host factors for all stages of its life cycle ^17^. The viral envelope is coated by spike protein trimers that bind to the host receptor angiotensin-converting enzyme 2 (ACE2), which is required for virus entry ^17– 19^. Host proteases, such as the transmembrane protease serine 2 (TMPRSS2), are also important as they cleave the spike protein, thus facilitating spike activation and subsequent viral entry ^18^.

Several studies have analysed transcriptomic ^20– 23^ and proteomic data (western blots, immunofluorescent microscopy ^24^ and immunohistochemistry data ^10^) to determine ACE2 expression levels in placental cells. Transcriptomic data demonstrates that placental expression of ACE2 is very low, while immunohistochemistry data has identified placental cells that express ACE2 protein. Placental TMPRSS2 expression is extremely low and not detectable in many placentas ^10, 20, 24^. The low levels of expression of both ACE2 and TMPRSS2 are important for ensuring the placenta, and thereby the foetus, are protected from infection. However, SARS-CoV-2 has been identified in placental tissue sections and although this is a rare event it is of serious concern ^9– 11, 24– 30^.

As viral transfer to the foetus typically does not occur, even in cases with evidence of placental infection ^24, 29– 32^, we sought to further develop our understanding of the interaction of placental cells with SARS-CoV-2.

Using transcriptomic data sets we analysed ACE2 expression, and found that it varies across placental cell types and gestation. However, analysis of ACE2 protein expression levels by confocal microscopy did not reveal a significant change in ACE2 expression from first trimester to term. *In vitro* assays determined that in line with their low ACE2 expression, only 0.5±0.15 % of trophoblasts can be infected with SARS-CoV-2 and they poorly support viral release. Other placental cell types are resistant to infection by SARS-CoV-2. We propose that these findings provide further understanding of the rare cases where SARS-CoV-2 can be found in trophoblast cells but ultimately, does not cross the placenta to infect the foetus *in utero*.

## Methods

### Patient samples

All tissue samples used were obtained with written informed consent from participants and collected under the Centre for Trophoblast (CTR) biobank permissions. Placentas were obtained from healthy women with apparently normal pregnancies undergoing elective first trimester terminations (6–12 weeks estimated gestational age (EGA) (n=20)). The EGA of the samples was determined from the last menstrual period. Peripheral blood from healthy volunteers was obtained with written informed consent from participants. Ethical approval was obtained from the Cambridge Research Ethics committee (study 04/Q0108/23). Term placentas were collected from healthy women with normal pregnancies and delivered by elective Caesarean section. Participants were consented for research sample collection as part of the surgical procedure, with further permission for storage and transfer of materials to the biobank given under REC ID 07/MRE05/44. Analysis was performed as part of the Cambridge Blood and Stem Cell Biobank REC ID 18/EE/0199. Samples are collected under the ethics of the CTR biobank and therefore ethical approval numbers are broad in scope and cover ongoing and past studies.

### Placenta digestion

Placentas were processed immediately upon receipt and as described previously ^33^. First trimester placentas were washed in PBS for 10 min with a stirrer before processing. The placental villi were scraped from the chorionic membrane with a scalpel and digested with 0.2 % Trypsin (Pan-Biotech, P10-025100P)/0.02 % Ethylenediaminetetraacetic acid (EDTA; Sigma-Aldrich, E5134) at 37°C with stirring, for 7 min. The digested cell suspension was passed through a sterile muslin gauze, and 5–10 ml heat-inactivated foetal bovine serum (Hi-FBS; Sigma-Aldrich, F9665-500ml) was added to halt the digestion process. The undigested tissue left on the gauze was scraped off with a scalpel and digested in 2.5 ml 1 mg/ml collagenase V (Sigma-Aldrich, C9263-1G), supplemented with 50 µl of 10 mg/ml DNAse I (Roche, 52779120) for 30 min at 37°C with agitation. The digested cell suspension was passed through a sterile muslin gauze and washed through with PBS. Cell suspensions from both the trypsin and collagenase digests were pelleted, resuspended in PBS and combined. Cell suspensions were layered onto a Pancoll gradient (PAN-biotech, P04-60500) and centrifuged for 20 min without brake at 1741 g. The leukocyte layer was collected and washed in PBS.

Primary trophoblast cells were isolated as previously described ^34^. Briefly, approximately 30–40 g of villous tissue were dissected free of decidua and blood vessels, washed in saline and digested in trypsin (0.25%, Invitrogen, Carlsbad, CA) and DNAse I (Sigma–Aldrich, St. Louis, MO). Digests were then poured through 70 μm cell filters (BD Bioscience, San Jose, CA) and cytotrophoblast cells purified over a discontinuous 10–70% Percoll gradient. Cells which migrated between 35–55% Percoll layers were collected and cultured in 1:1 mixture of Dulbecco’s modified Eagle’s medium (DMEM, Sigma–Aldrich) and Ham’s F-12 nutrient mixture (Invitrogen) containing 10% fetal bovine serum (FBS, Atlanta Biologicals, Lawrenceville, GA), 50 μg/ml gentamicin, 60 μg/ml benzyl penicillin and 100 μg/ml streptomycin (Sigma–Aldrich). Cells were cultured as described below.

### Immunofluorescence of placental tissue sections

First trimester and term placenta villous tissue sections were prepared as previously described ^35^. Briefly, slides were fixed and permeabilized by submersion in ice-cold acetone for 5 min and washed in PBS. Slides were placed in blocking buffer at room temperature for 20 min and incubated overnight at 4°C with primary antibodies ( Table 1). Slides were washed twice in PBS and incubated with secondary antibodies for 1 h at room temperature in the dark. Where conjugated antibodies were used, these were added together with the secondary antibodies. Slides were washed twice in PBS and stained with DAPI (Sigma-Aldrich, D9542; diluted 1:1000 in PBS) for 5 min in the dark, washed twice in PBS and mounted using Ibidi Mounting Medium (Ibidi, 50001). Slides were imaged using the Zeiss SP8 confocal LSM 700 and the Zeiss LSM 780 AxioObserver.

**Table 1.** Antibodies for microscopy.

RRIDs	Antibody	Species	Source	Identifier
**-**	DAPI	-	Sigma-Aldrich	D9542
**-**	Hoechst 33342	-	Thermo Fisher Scientific	62249
**AB_301861**	Anti-ACE2 (unconjugated; polyclonal)	Rabbit	Abcam	ab15348
**AB_2134589**	Anti-human cytokeratin 7 (unconjugated; clone OV- TL 12/30, monoclonal)	Mouse	Dako	M7018
**AB_2864418**	Anti-SARS-CoV/SARS-CoV-2 (COVID-19) spike protein (unconjugated; clone 1A9, monoclonal)	Mouse	GeneTex	GTX632604
**AB_2566326**	Anti-human CD31 (AF700; clone WM59, monoclonal)	Mouse	Biolegend	303133
**AB_143165**	Goat anti-rabbit IgG (H+L) AF488 (polyclonal)	Goat	Thermo Fisher Scientific	A-11008
**AB_2762823**	Donkey anti-mouse IgG (H+L) AF488 (polyclonal)	Donkey	Thermo Fisher Scientific	A-32766
**AB_2536180**	Donkey anti-mouse IgG (H+L) AF555 (polyclonal)	Donkey	Thermo Fisher Scientific	A-31570

For quantification of ACE2 expression in placental sections, confocal microscopy was performed using the Zeiss LSM 780 AxioObserver using the 40x objective. Four images at a single z-plane were acquired per donor at 2048 × 2048 pixels and 0.64 μs pixel dwell time. Binary images of the endothelium (CD31) and trophoblasts (cytokeratin 7; CK7) were converted into masks using the particle analysis function in the Fiji software ^36^ and an inverted selection created for cell-specific quantification of ACE2 expression. Selections were overlaid over a binary image of ACE2 expression (set to a threshold of 1100-65535) and the percentage area of positive expression quantified per image. Of note, to create the trophoblast-specific selection, CD31 endothelium selection was deleted from the CK7 mask prior to creating the inverted selection due to the spillover of the CD31 AF700 channel into the CK7 AF555 channel. For representative images, cells were imaged using the 40x objective a Zeiss LSM 780 AxioObserver. Images were cropped from 2048 × 2048 pixels to 980 × 951 pixels and scale increased from 9.635 pixels/µm to 13.764 pixels/µm. For magnified panels, images were further cropped to 310 × 310 pixels.

### Culture of cell lines and primary placental cells

HeLa and HeLa + ACE2 cells were a gift from Dr. James Edgar, University of Cambridge, UK ^37^. JEG-3 and JAR choriocarcinoma cell lines obtained from American Type Culture Collection (ATCC) were a gift from Prof. Ashley Moffett ^38^. HUVECs were a gift from Dr. Luz Alonso-Crisostomo and Prof. Nick Coleman (Sigma-Aldrich, 200P-05N, pooled donors). All cell lines and primary cells, bar HUVECs, were cultured in advanced DMEM/F-12 (Thermofisher Scientific, 12634-010) supplemented with 10 % Hi-FBS, 10 mM L-glutamine (Thermofisher Scientific, 25030024), 10 U/ml Pen/Strep (Gibco, 15140-122), 2.5 μg/ml Amphotericin B (Sigma-Aldrich, A9528) and 0.5 µg/ml Gentamycin (Sigma-Aldrich, G1272) in 5 % CO _2_ at 37°C. HUVECs were maintained in Endothelial Cell Growth Medium (Cell Applications, Inc, 211-500) supplemented with 2 % Hi-FBS and antibiotics as described above. Primary trophoblast cells were maintained in culture with daily media changes. For generating moMacs, human peripheral blood mononuclear cells (PBMCs) were isolated using a density gradient centrifugation with Pancoll (PAN-Biotech, P04-60500). Monocytes were then isolated using the MojoSort™ Human Pan Monocyte Isolation Kit performed according to the manufacturer’s instructions (Biolegend, 480060) and cultured in media supplemented with M-CSF (Gibco, PHC9501) with further 50 % media changes at 3 and 5 days of culture. Human primary HBCs were isolated as previously described ^35^, briefly by cell sorting using a BD FACS Aria III (BD Biosciences) and selecting CD45 ^+^, CD14 ^+^ and HLA-DR ^-^ cells. HBC were cultured in media supplemented with M-CSF with further 50 % media changes at 3 and 5 days of culture. Primary placental fibroblasts were obtained by culturing first trimester placental digests with 20 % Hi-FBS for 2–3 weeks.

### Viruses

The BetaCoV/England/MIG457/2020 (B.1.1.7) strain of SARS-CoV-2 [B.1.1.7] was obtained from Public Health England (Colindale), in January 2021 and was passaged twice on VeroE6+ACE2+TMPRSS2 cells to generate the stocks used in this study. Virus sequences were verified by next generation sequencing ^39^ and no mutations were identified in the stocks compared to the parental sequences.

### SARS-CoV-2 infections

HeLa, HeLa + ACE2, JEG-3 and JAR cells, HUVECS, moMacs, primary fibroblasts, primary trophoblasts, primary first trimester and term HBC were plated on to 96-well glass-bottomed plates (Eppendorf, 00-30-741030). Cell lines (HeLa, HeLa + ACE2, JEG-3 and JAR cells, HUVECS) were seeded at 10,000 cells/well the day before infection. Primary fibroblasts were seeded at 10,000 cells/well two days prior to infection. Due to the nature of the cells primary trophoblasts were seeded at a 20x higher density of 200,000 cells/well up to three days prior to infection. Similarly, moMacs and HBC were seeded at a 10x higher density of 100,000 cells one week prior to infection. Cells were washed once with PBS before incubation with stock virus diluted in sera-free media (MOI 1), for 1 h at room temperature on a rocking platform. After the inoculum was removed, cells were washed with PBS, and 100 μL of the relevant media containing 2 % fetal bovine serum was added. At 24 hpi, cell monolayers were fixed with 4 % paraformaldehyde (Thermofisher Scientific, J19943.K2). PFA was quenched with 2×5 min washes with 20 mM glycine (Melford, G36050-1000.0)/PBS prior to proceeding to immunofluorescence staining and imaging.

To assess virus release from infected placental cells, the cells were washed with PBS following infection as above. 100 μl of media (2 % fetal bovine serum) was added to each well and the cells were incubated under standard conditions for 30 min. This media was harvested and replaced with 100 μl of fresh media, which was harvested at 24 hpi. Both the 30 minute post-infection (mpi) and 24 hpi virus samples were stored at -80°C until titration.

### Plaque assays

VeroE6 + ACE2 + TMPRSS2 cells were kindly provided by the University of Glasgow ^40^ and were cultured under standard conditions (37°C and 5 % CO _2_) in high-glucose DMEM (Thermofisher Scientific, 11965092) supplemented with 10 % heat-inactivated fetal bovine serum (Gibco, 10500-064), 100 units/ml penicillin, 100 µg/ml streptomycin (Pen/Strep, Gibco, 15070063), 2 mM L-glutamine (Gibco, 25030081), 25 mM HEPES (Merck, H0887-100ML), 1 % non-essential amino acids (Gibco, 11140050), 2 µg/ml hygromycin B (Roche, 10843555001) and 2 mg/ml G418 (Gibco, 11811031). The cells for viral passage and plaque assay were regularly tested and confirmed to be free of mycoplasma (MycoAlert Mycoplasma Detection Kit, Lonza, LT07-710).

For viral titration by plaque assay, VeroE6+ACE2+TMPRSS2 cells were seeded into 6 well plates at 40 % confluency the day before infection. Virus samples were serially diluted into sera-free media, which was added to PBS-washed cells. Infections were conducted on a rocking platform for 1 h at room temperature. Cells were washed with PBS before an overlay of media containing 2 % FBS and 0.2 % low-melt point agarose (Thermofisher Scientific, 16520050) was added to the monolayer. At 72 hpi, cells were fixed with 10 % neutral-buffered formalin (Sigma-Aldrich, HT501128-4L) for 1 h. Fixed monolayers were stained with toluidine blue (Sigma-Aldrich, 89640), washed and the plaques were counted manually.

### Whole-mount immunofluorescence and microscopy for SARS-CoV-2 infections

Cells were permeabilized with 0.1 % saponin (Sigma-Aldrich, S7900-2G)/PBS for 10 min and subsequently blocked in 1 % Bovine Serum Albumin (BSA; Thermofisher Scientific, BP9702-100), 0.01 % saponin in PBS for 20 min. Cells were incubated with primary antibodies diluted in blocking solution for 1 h at room temperature and washed 3×5 min in blocking solution. Secondary antibodies diluted in blocking solution were added and incubated in the dark for 45 min at room temperature. After 3×5 min washes with blocking buffer, cells were stained with Hoechst 33342 (Thermo Fisher Scientific, 62249; diluted 1:1000 in PBS) for 5 min in the dark and washed twice more in PBS before placing in PBS prior to imaging. For quantification, cells were imaged using the 20x objective on a Zeiss LSM 780 AxioObserver. Three images were acquired per uninfected well of each cell type. Five to six images were acquired for each infected well and the total number of cells and the number of infected cells counted manually using the multipoint tool in Fiji. At least 44 cells were counted per well from 4 independent experiments. For representative images, cells were imaged using the 40x objective a Zeiss LSM 780 AxioObserver. Images were cropped from 3168×3168 to 2156×2156.

### Analysis of publicly available scRNAseq data

scRNAseq data of first trimester placenta ^41^ were obtained from EMBL-EBI ArrayExpress ( https://www.ebi.ac.uk/arrayexpress) under the experiment code E-MTAB-6701. scRNAseq data of full-term placenta ^42^ were obtained upon request from lead authors of the study. Sequencing data from placental samples were aligned using the Cell Ranger Single-Cell Software Suite (v3.0; 10x Genomics) against the GRCh38.93 human reference genome. Downstream analysis of each sample was performed using Seurat (v3.0 ^43^). Cells with <500 detected genes and >20 % mitochondrial gene expression were removed. Samples were log-normalized and integrated following the Seurat v3 Integration workflow. Clusters were identified using the FindNeighbors and FindClusters functions in Seurat. Clusters were annotated on the basis of expression of known marker genes. UMAP dimensionality reduction was performed using the RunUMAP function in Seurat, with default parameters.

Annotated scRNAseq datasets from the small intestine ^44^, nasal mucosa, bronchi, and lung parenchyma ^45^ were downloaded from the COVID-19 Cell Atlas repository ( https://www.covid19cellatlas.org/). Each dataset was loaded into R and subjected to the same Seurat workflow as detailed above starting from the sample integration step. Annotations from the original studies were then super-imposed onto the resultant UMAP embeddings. Due to the sparsity observed in scRNAseq data, and the generally low expression levels of expression of *ACE2* and *TMPRSS2* across tissues, we used the Nebulosa package in R ^40^ to visualise kernel density estimates of expression and co-expression across datasets, allowing for better interpretation of low-level expression across datasets. Gene expression dot plots of log-normalised gene expression of *ACE2* and *TMPRSS2* were generated for each dataset using the DotPlot function in Seurat. The data was extracted for each plot, merged and plotted as a single dot plot for all combined datasets using ggplot.

## Results

### Trophoblast cells express similar levels of ACE2 transcript as adult airway cells

**Figure 1.**
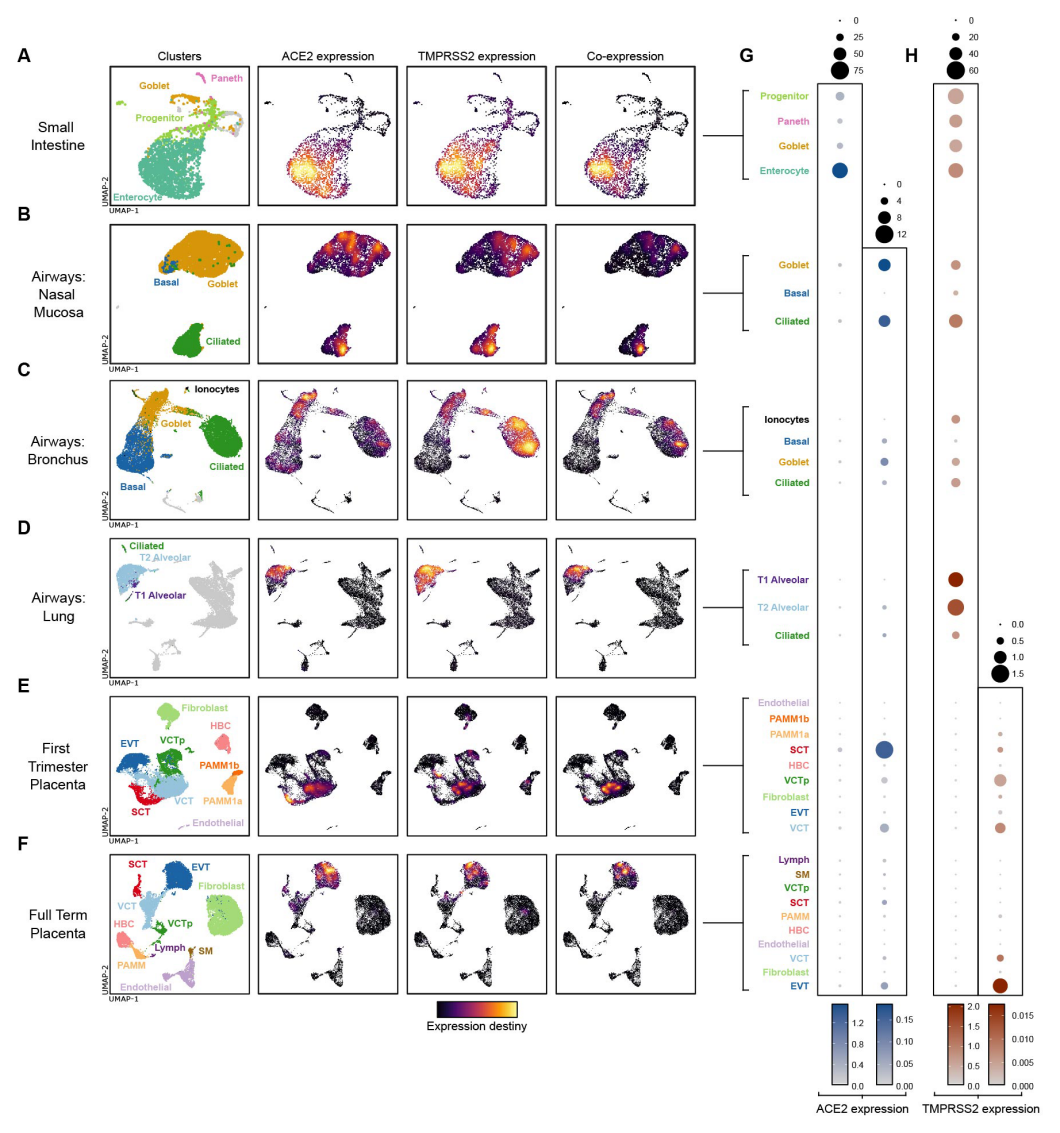
ACE2 and TMPRSS2 gene expression in placental scRNAseq datasets. ( **A**– **F**) UMAP visualisation and Nebulosa kernel density estimates of ACE2 expression, TMPRSS2 expression and ACE2-TMPRSS2 co-expression of single cells from the human **(A)** small intestine ^44^, **B**) nasal mucosa, ( **C**) bronchi, ( **D**) lung parenchyma and alveoli ^45^, ( **E**) first trimester placenta ^41^ and ( **F**) term placenta ^42^. Left-hand plots – cells are coloured by their cluster identity. In the small intestine, nasal mucosa, bronchi, and lung parenchyma datasets, cells with no expression of either ACE2 or TMPRSS2 are coloured grey. ( **G**, **H**) Dot plot heatmaps displaying log-normalised gene expression of ( **G**) *ACE2* and ( **H**) *TMPRSS2* across different datasets and tissues. Two different scales are used for each gene, to compare ACE2 and TMPRSS2 expression both within and between different tissues. Dot size represents fraction of cells with nonzero expression.

Previous studies have indicated that placental cells express the SARS-CoV-2 entry receptor ACE2, albeit at low levels ^20, 21^. We sought to further understand placental cell expression of ACE2 and TMPRSS2 across gestation, and how it compares with the small intestine and airways, which are primary sites of SARS-CoV-2 infection in adults ^46– 48^. To do this we integrated publicly available datasets of the human small intestine ^44^, nasal mucosa, bronchi, lung parenchyma, alveoli ^45^, first trimester ^41^ and term placenta ^42^. UMAP visualisation and Nebulosa kernel density estimates provide insight into *ACE2*, *TMPRSS2* expression and *ACE2-TMPRSS2* co-expression across these tissues ( Figure 1A–F). In agreement with work of others, dot plot heatmaps displaying log-normalised gene expression demonstrate that, of all the tissues profiled, *ACE2* and *TMPRSS2* are most highly expressed in small intestine enterocytes ^49, 50^. Within the airways, ciliated and goblet cells of nasal mucosa express the highest levels of *ACE2* and *TMPRSS2* ^20, 48, 51^ ( Figure 1G, H). In the placenta, *ACE2* is most highly expressed in first trimester syncytiotrophoblast (SCT). Surprisingly, first trimester SCT express similar levels of *ACE2* as cells of the nasal airway mucosa. *ACE2* expression by term placental cells is extremely low ( Figure 1G). Both first trimester and term placental cell expression of *TMPRSS2* is very low, in comparison with cells of the small intestine and airways ( Figure 1 H).

### Placental ACE2 protein expression

**Figure 2.**
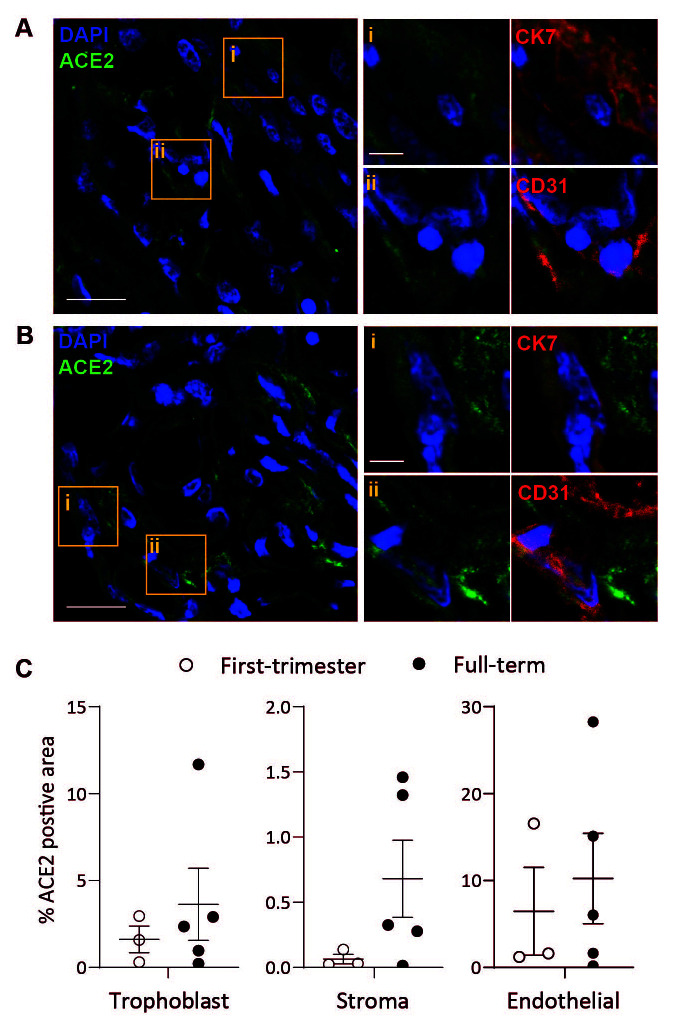
Placental cell expression of ACE2. Representative confocal microscopy images of first trimester ( **A**) and term ( **B**) placental villi sections stained for ACE2 (green) and DAPI (blue). Boxes in the left main image highlight ACE2 expression that colocalise with ( **i**) Cytokeratin 7 (CK7, red, shown magnified top right panels) or ( **ii**) CD31 (red, shown magnified bottom right panels). Merged magnified images with CK7 or CD31 (red) are shown on the far right. Scale bar, 20 µm (main) and 5 µm (inset). ( **C**) Quantification of ACE2 staining shown as a percentage of the total area of CK7 ^+^ (trophoblast), CD31 ^+^ (endothelial) or CK7 ^-^ CD31 ^-^ (stromal) staining in first trimester (n=3, open-circle) or term (n=5, closed circle) placental sections. Significance calculated using Mann-Whitney test; all first trimester and term comparisons in all cell types are not significant, p>0.05.

Transcriptomic analysis is not always representative of protein expression, hence we sought to further resolve ACE2 expression by placental cells across gestation. In agreement with work by others ^10^, using immunofluorescence microscopy, we find low levels of variable ACE2 expression across a range of placental cell types, including trophoblast (cytokeratin 7 ^+^), endothelial (CD31 ^+^), and stromal cells ( Figure 2A, B). Quantification of ACE2 positive staining in trophoblast, stromal and endothelial cells showed, there is a trend, but no significant change in ACE2 protein expression from first trimester to term ( Figure 2C).

### Placental cells are resistant to SARS-CoV-2 infection

We sought to determine if the levels of ACE2 cell surface expression we detected on placental cells are sufficient to support SARS-CoV-2 infection, RNA replication and protein translation and virion assembly and release. For this assay, we focused on term placental trophoblast cells, due to their trend for higher levels of ACE2 expression than first trimester trophoblast cells. We infected primary trophoblast cells, fibroblasts and HBC isolated from term placentas with SARS-CoV-2. We included HUVECs, as a representative of placental endothelial cells, the commonly used placental cell lines JEG-3 and JAR and monocyte-derived macrophages (moMacs). HeLa cells were lentivirally transfected to stably express ACE2 (HeLa + ACE2) and were used as a positive control. Parental HeLa (HeLa) cells were used as a negative control.

**Figure 3.**
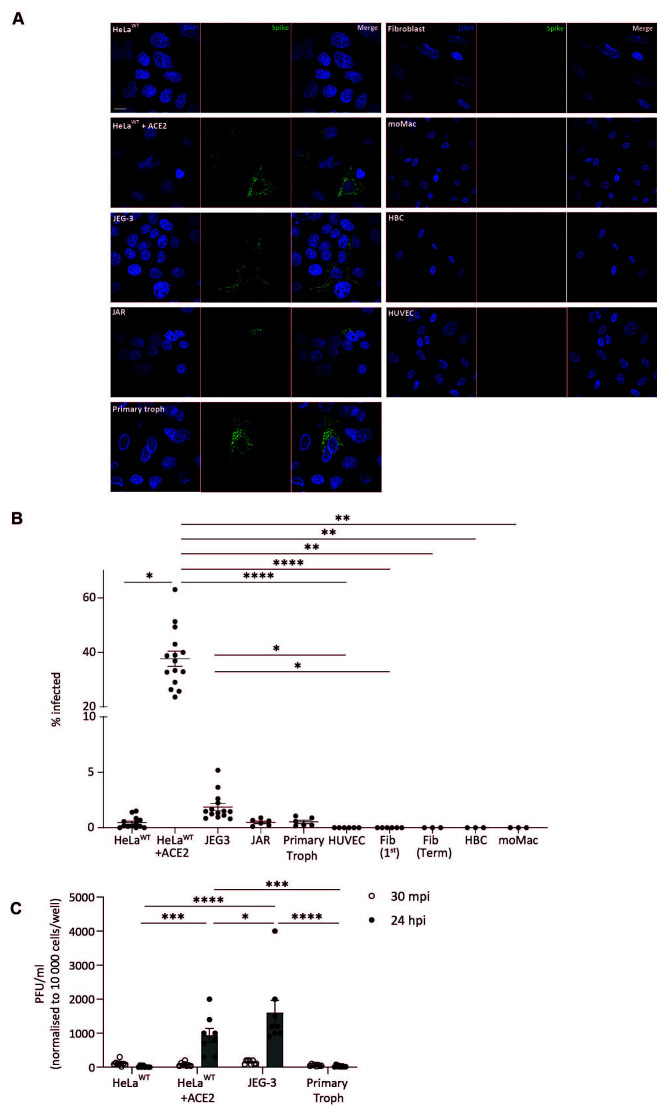
SARS-CoV-2 infection and subsequent replication within primary trophoblasts is minimal. ( **A**) Representative microscopy images of cells infected with SARS-CoV-2 (MOI 1) and fixed at 24 hours post-infection (hpi). HeLa ^WT^ and HeLa ^WT^+ ACE2 cells were used as negative and positive infection controls respectively. Cells are stained for spike protein (green, middle panels) and Hoechst 33342 (blue, left panels) and merged images are shown (right panels). Scale bar, 20 µm. ( **B**) Quantification of the percentage of spike protein positive cells for indicated cells infected with SARS-CoV-2 (MOI 1) and fixed at 24 hpi. Pooled data from at least one independent experiment with at least three wells per cell type. Mean±s.e.m. shown. Significance calculated using one-way ANOVA with Kruskal-Wallis multiple comparisons test. * p<0.05, **, 0.01, **** p<0.0001. ( **C**) Supernatants from infected cells were collected 30 minutes post-infection (mpi; open circles, white bars) and 24 hpi (closed circles, grey bars). The viral titre from indicated cells are shown as PFU/ml normalised to 10 000 cells/well. Pooled data from two independent experiments with at least three wells per cell type. Mean±s.e.m. shown. Significance calculated using two-way ANOVA with Tukey’s multiple comparisons test. * p<0.05, ***, 0.001, **** p<0.0001.

We find that our positive controls, HeLa + ACE2 cells are readily infected with SARS-CoV-2, with 37.7±2.77 % of cells infected. In comparison, parental HeLa cells are not permissive to infection, with 0.5±0.14 % of the cells infected ( Figure 3A, B). We find that HUVECs, primary placental fibroblasts, HBC and moMacs are completely resistant to infection with SARS-CoV-2 ( Figure 3A). JEG-3 cells, the choriocarcinoma trophoblast cell line, are more permissive to infection than JAR cells (1.9±0.33 % and 0.5±0.12 % respectively). Despite the significantly lower infection rate of JEG-3 cells compared to the HeLa + ACE2 positive control, PFU virion release by JEG-3 cells is significantly higher than both controls and any other tested cell type ( Figure 3C). These data show that while JEG-3 cells are less permissive to viral entry they are highly permissive to viral replication and virion release. In contrast, primary term trophoblast cells show infection levels similar to the parental HeLa negative control (0.5±0.15 %; Figure 3B) but show marginally higher levels of PFU virion release than parental HeLa cells ((29.5±10.30 and 17.5±10.48 PFU/ml respectively); Figure 3C)). This suggests that the surface expression of ACE2 and intracellular environment of primary trophoblast cells make them minimally permissive to both viral infection and replication, and thus would not facilitate viral spread or cell-to-cell transmission.

## Discussion

In this study we sought to understand discrepancies in the field, where congenital infection typically does not occur when the mother is infected with SARS-CoV-2 ^14^, yet SARS-CoV-2 has been identified in placental tissue sections. Using transcriptomic data sets, we find that the expression of the SARS-CoV-2 entry receptor ACE2 by all placental cells is extremely low but similar to adult airway cells. TMPRSS2 expression by placental cells is negligible. The lack of TMPRSS2 implies that placental cells may be protected from SARS-CoV-2 infection. However, blocking TMPRSS2 activity only partially inhibits SARS-CoV-2 infection ^18^, suggesting a potential role for other proteases such as furin ^52^ and cathepsin L ^18^ in aiding viral transmission ^53^; both are expressed by placental cells ^54– 56^.

Hence, using *in vitro* infection assays we sought to determine if different placental cell types are permissive to SARS-CoV-2 infection. HUVECs, HBC and placental fibroblasts showed no evidence of SARS-CoV-2 infection, in line with their negligible expression of ACE2. In contrast, we find that 0.5 ± 0.15 % of primary term trophoblast cells are permissive to SARS-CoV-2, albeit at low levels. In addition, primary trophoblast cells only support very low levels of viral release. JEG-3 and JAR cell lines poorly recapitulate trophoblast biology, indicating they are a poor model for understanding the interactions of SARS-CoV-2 with the placenta. These findings agree with the conclusions of others ^57^ and suggest that the ability of SARS-CoV-2 to infect trophoblast cells and spread across the placenta to the foetus is impaired.

## Ethics and consent

Ethical approval was obtained from the Cambridge Research Ethics committee (study 04/Q0108/23). Term placentas were collected from healthy women with normal pregnancies and delivered by elective Caesarean section. Participants were consented for research sample collection as part of the surgical procedure, with further permission for storage and transfer of materials to the biobank given under REC ID 07/MRE05/44. Analysis was performed as part of the Cambridge Blood and Stem Cell Biobank REC ID 18/EE/0199. Samples are collected under the ethics of the CTR biobank and therefore ethical approval numbers are broad in scope and cover ongoing and past studies.

## Data Availability

Apollo - University of Cambridge Repository: Human placental cells and SARS-CoV2, https://doi.org/10.17863/CAM.106485 ^58^. The repository contains the following data: Flow cytometry - Fcs file: Data used to isolate HBC from placental digests using the BD FACSDiva™ software on the BD FACS Aria III. Related to Figure 3. Microscopy – Tif files: First-trimester villi section stained for ACE2 (green), cytokeratin 7 (red) and CD31 (far red) to visualise ACE2 expression in trophoblast, endothelial and stromal compartments. Sections were imaged on the Zeiss LSM 780 using the 40x objective. Related to Figure 2A. Term villi section stained for ACE2 (green), cytokeratin 7 (trophoblast, red) and CD31 (endothelium, far red) to visualise ACE2 expression in trophoblast, endothelial and stromal compartments. Sections were imaged on the Zeiss LSM 780 using the 40x objective. Related to Figure 2B. Confocal images of the different cell types (specified in the file name) infected with SARS-CoV-2 (MOI 1) and fixed at 24 hours post-infection (hpi). Cells are stained for nuclei (blue) and spike protein (green) and imaged on the Zeiss LSM 780 using the 40x objective. Related to Figure 3A. Data are available under the terms of the Creative Commons Attribution 4.0 International license (CC-BY 4.0). scRNAseq data of first trimester placenta ^41^ were obtained from EMBL-EBI ArrayExpress ( https://www.ebi.ac.uk/arrayexpress) under the experiment code E-MTAB-6701. scRNAseq data of full-term placenta ^42^ were obtained upon request from lead authors of the study. Annotated scRNAseq datasets from the small intestine ^44^, nasal mucosa, bronchi, and lung parenchyma ^45^ were downloaded from the COVID-19 Cell Atlas repository ( https://www.covid19cellatlas.org/).
