# Supplementary material for: Human placental cells are resistant to SARS-CoV-2 infection and replication
Source: Wellcome Open Res. 2024 Nov 22;9:209. Originally published 2024 Apr 18. [Version 2] doi: 10.12688/wellcomeopenres.20514.2 (PMC11617822; doi:10.12688/wellcomeopenres.20514.2)
Supplement: Peer review history

# Reviewer report: Reviewer response for version 2

**DOI:** 10.21956/wellcomeopenres.25762.r115777

**Published:** 2025-01-02

**Authors:** Megan C Sharps

**Affiliations:** 1 The University of Manchester, Manchester, England, UK

The authors have used single cell RNA sequencing of human placenta, alongside additional samples such as bronchi, lung parenchyma and nasal mucosa to map expression of *ACE2 *and *TMPRSS2*. Immunofluorescence of placental sections to detect the ACE2 protein was performed. Finally, placental cells were infected with SARS-CoV-2 to determine how easily the virus can infect and replicate within each cell type. There are clear figures, and the discussion section covers the limitations of the study, including possible differences between SARS-CoV-2 strains and the cell lines used in the experiments.

**I have a few minor comments on the article:**

**Abstract:**

- Whilst further work is not carried out with TMPRSS2, there is no mention of investigating its expression in the abstract. This could be added to cover the entire research conducted in the scRNAseq datasets.

**Introduction:**

- In the second paragraph of the introduction, fetus should be corrected to foetus for continuity.
- Also in the second paragraph the text reads *"It is a highly effect barrier"*. Should this read *"It is a highly effective barrier"*?

**Methods:**

- For ease of reading, can moMacs be defined in the methods for those unused to this acronym.
- It would be beneficial for HBCs to be defined after the first use of Hofbauer Cells.
- Whilst mpi has been defined in the methods, it would be helpful if hpi could be defined in the *SARS-CoV-2 infections *section of the methods.

**Results:**

- For those unfamiliar with the different cell types in the human placenta, definitions of  the acronyms in Figure 1 such as PAMM, VCTp and SMC would be beneficial in the legend.
- Were the supernatants for all the cell types displayed in Figure 3b analysed? Only a subset of data has been presented in Figure 3c. Whilst the percentage of infected cell is low in HUVECs, fibroblasts, HBCs and moMacs, JAR cells had comparative values to HeLa and primary trophoblasts. However, JAR cells have not been included in the supernatant analysis.  It would be beneficial for the article if these data were included if the data were available.

Is the work clearly and accurately presented and does it cite the current literature?

Yes

If applicable, is the statistical analysis and its interpretation appropriate?

Yes

Are all the source data underlying the results available to ensure full reproducibility?

Partly

Is the study design appropriate and is the work technically sound?

Yes

Are the conclusions drawn adequately supported by the results?

Yes

Are sufficient details of methods and analysis provided to allow replication by others?

Yes

Reviewer Expertise:

Placental biology, single cell RNA sequencing, inflammation, macrophages, effect of SARS-CoV-2 on placenta.

I confirm that I have read this submission and believe that I have an appropriate level of expertise to confirm that it is of an acceptable scientific standard.

# Reviewer report: Reviewer response for version 2

**DOI:** 10.21956/wellcomeopenres.25762.r113127

**Published:** 2024-12-04

**Authors:** Takada Kazuhide

**Affiliations:** 1 Nihon University School of Medicine, Tokyo, Japan

In the presented study, Yoshida and colleagues investigated the ACE2 and TMPRSS2 gene expression using placental scRNAseq datasets and immunofluorescence techniques. They also performed in vitro experiments to evaluate the infection of SARS-CoV-2 employing primary trophoblast cells, fibroblasts and Hofbauer cells, and trophoblast cell lines. They showed that ACE2 positive staining in trophoblast, stromal and endothelial cells, and no significant change in ACE2 protein expression from first trimester to term. They also demonstrated that SARS-CoV-2 could infect primary trophoblasts, but subsequent replication is minimal.

 It is interesting that SARS-CoV-2 can infect the placenta but vertical infection is very rare. The presented study is very important because it attempts to clarify part of this mechanism. There are also no obvious shortcomings in the methodology. However, the authors should address some points mentioned below before the study is completely approved.

**Major points:**

 1. The citations are insufficient. It has already been reported that JEG-3 cells are resistant to SARS-CoV-2 infection Tallarek *et.al* (2021 ^1^ ). An “abortive infection” of SARS-CoV-2 in the placenta and a conflicting report have already been published Takada *et.al *(2022 ^2^ ) and Tiozzo *et.al* (2024 ^3^ ). These previous studies do not eliminate the value of the presented study because, as the authors point out, differences in virus strains may have an impact. However, it is unfair not to mention these studies. The authors should conduct a careful literature search and discuss these publications.

 2. scRNAseq and immunofluorescence in the absence of infection provides us with a variety of information. However, a previous study showed that the levels of ACE2 immunoreactivity were significantly higher in trophoblastic cells of fibrin-encased villi than in those of free-floating villi Mao *et.al* (2022 ^4^ ). Maternal SARS-CoV-2 infection with or without placental infection may cause coagulation in the placenta Victoria M (2022 ^5^ ), which may elevate ACE2 expression in the placenta and lead to subsequent SARS-CoV-2 infection in the placenta. Interestingly, the infected placentas may reduce ACE2 expression Verma *et.al* (2021 ^6^ ). The reviewer thinks consideration of changes in ACE2 expression during infection in addition to the placental expression of ACE2 in usual conditions would make this study more informative for the readers.

**Minor point:**

 3. Does “HBC” represent Hofbauer cell? Please describe what HBC stands for.

Is the work clearly and accurately presented and does it cite the current literature?

No

If applicable, is the statistical analysis and its interpretation appropriate?

Yes

Are all the source data underlying the results available to ensure full reproducibility?

Yes

Is the study design appropriate and is the work technically sound?

Yes

Are the conclusions drawn adequately supported by the results?

Partly

Are sufficient details of methods and analysis provided to allow replication by others?

Yes

Reviewer Expertise:

Microbiology, Reproductive Immunology.

I confirm that I have read this submission and believe that I have an appropriate level of expertise to confirm that it is of an acceptable scientific standard, however I have significant reservations, as outlined above.

## References

[1] : Inefficient Placental Virus Replication and Absence of Neonatal Cell-Specific Immunity Upon Sars-CoV-2 Infection During Pregnancy. *Front Immunol* .2021;12: 10.3389/fimmu.2021.698578 698578 10.3389/fimmu.2021.698578 34149740 PMC8211452

[2] : Restriction of SARS-CoV-2 replication in the human placenta. *Placenta* .2022;127: 10.1016/j.placenta.2022.07.010 73-76 10.1016/j.placenta.2022.07.010 35973367 PMC9293377

[3] : Placental SARS-CoV-2 viral replication is associated with placental coagulopathy and neonatal complications. *American Journal of Obstetrics and Gynecology* .2024;230(4) : 10.1016/j.ajog.2023.11.1222 e33-e37 10.1016/j.ajog.2023.11.1222 37952868 PMC10990825

[4] : Placental SARS-CoV-2 distribution correlates with level of tissue oxygenation in COVID-19-associated necrotizing histiocytic intervillositis/perivillous fibrin deposition. *Placenta* .2022;117: 10.1016/j.placenta.2021.12.002 187-193 10.1016/j.placenta.2021.12.002 34929459 PMC8638245

[5] : SARS-CoV-2 infection and COVID-19 vaccination in pregnancy. *Nat Rev Immunol* .2022;22(5) : 10.1038/s41577-022-00703-6 277-282 10.1038/s41577-022-00703-6 35304596 PMC8931577

[6] : SARS-CoV-2 colonization of maternal and fetal cells of the human placenta promotes alteration of local renin-angiotensin system. *Med* .2021;2(5) : 10.1016/j.medj.2021.04.009 575-590.e5 10.1016/j.medj.2021.04.009 33870242 PMC8043616

# Reviewer report: Reviewer response for version 2

**DOI:** 10.21956/wellcomeopenres.25762.r112957

**Published:** 2024-12-04

**Authors:** Joan Röhl

**Affiliations:** 1 Faculty of Health Sciences and Medicine, Bond University, Robina, Queensland, Australia

I have reviewed the new version of the article and consider the revisions as appropriate. I believe they are sufficient for to amend the previous status of 'Approved with reservations' to 'Approved'.

Is the work clearly and accurately presented and does it cite the current literature?

Partly

If applicable, is the statistical analysis and its interpretation appropriate?

Yes

Are all the source data underlying the results available to ensure full reproducibility?

Partly

Is the study design appropriate and is the work technically sound?

Yes

Are the conclusions drawn adequately supported by the results?

Partly

Are sufficient details of methods and analysis provided to allow replication by others?

Yes

Reviewer Expertise:

Cell biology, immunofluorescence microscopy, cancer biology, prostate cancer, bone metastasis, 3D cell culture models, inflammation, macrophages, gene expression, histology, vertical transmission of SARS-CoV-2, placenta biology, wound healing, intracellular trafficking, MMP14, cell migration

I confirm that I have read this submission and believe that I have an appropriate level of expertise to confirm that it is of an acceptable scientific standard.

# Reviewer report: Reviewer response for version 1

**DOI:** 10.21956/wellcomeopenres.22708.r85923

**Published:** 2024-07-19

**Authors:** Joan Röhl

**Affiliations:** 1 Faculty of Health Sciences and Medicine, Bond University, Robina, Queensland, Australia

**Summary of the research and overall impression:**

 The research article “ *Human placental cells are resistant to SARS-CoV-2 infection and replication*” is an interesting, well-designed, small study that adds to our understanding of why vertical transmission of SARS-CoV-2 does not occur frequently. The authors determined cellular ACE2 expression (primarily) on RNA and protein level within first trimester and term placental tissue and tested SARS-CoV-2 infection for various placental cell types in an *in vitro* infection assay. Their findings support the discourse and existing evidence as to why vertical transmission of SARS-CoV-2 is rare.

**Major concerns:**

 This is a well-designed study with a clear methodology. Unfortunately, the abstract does not do justice to the work done. Most strikingly, there is a disconnect between the methods section of the abstract (which only refers to the scRNAseq), the results section of the abstract (which only talks about the infection assay findings), and the respective section in the main body of the article. Redrafting the abstract to make sure it is reflective on the full breadth of the study is highly advised.

 For the placental ACE2 protein expression analysis via immunofluorescence microscopy, the data shown would be more compelling if the study used a positive and negative control, for example the HeLa+ACE2 and HeLa WT cell lines used in other parts of the study. At a minimum, the raw microscope files (rather than just the tiff image files) could be provided.

 Considering the work performed and the findings made in this study, the discussion appears to be too brief, lacking to address important points, and thereby underselling the research. The following points should/could be covered to give the discussion more depth:

- While differences in RNA vs. protein expression are common, could this have any biological meaning for the observations made in this study?
- What could be the cause of discrepancies in infection rate vs virion release in JEG3 vs primary trophoblasts (other than just calling them a “poor model”)? Can you also put the findings on infection rates of the other cell types investigated into the context of the literature?
- What implications (if any) does the use of the specific SARS-CoV-2 strain used in this study have on the findings? Would using a different strain change any of the findings? Why/why not?
- What are the limitations of the study? Future directions?

**Minor suggestions:**

 Depending on the audience the authors hope to target, a paragraph on placental architecture, structures, and cell types would ensure that a wide range of readers have all relevant background information required to fully appreciate the study findings.

 To further add to the data availability, it could be helpful to provide (exemplary) images of the CD31 / CK7 masks that were used to quantify ACE2 protein expression in the immunofluorescence of placental tissues.

 Please indicate the type of error bar that is presented in Figure 2C in the figure legend.

 In the results section “Placental cells are resistant to SARS-CoV-2 infection”, the specific reference to protein expression could be made to further strengthen the rationale for aspects of the infection assay: “ *For this assay, we focused on term placental trophoblast cells, due to their trend for higher levels of ACE2 [ **protein**] expression than first trimester trophoblast cells.*”

 In Figure 3A, the figure legend indicates that Hoechst stain was used, but in the figure itself, it labels the blue channel as “DAPI”.

 In Figure 3B and C, it might be better to remove the “WT” superscript from “HeLa(WT)+ACE2”.

**Conclusion:**

 I support this manuscript for indexation after the authors suitably address the comments made above.

Is the work clearly and accurately presented and does it cite the current literature?

Partly

If applicable, is the statistical analysis and its interpretation appropriate?

Yes

Are all the source data underlying the results available to ensure full reproducibility?

Partly

Is the study design appropriate and is the work technically sound?

Yes

Are the conclusions drawn adequately supported by the results?

Partly

Are sufficient details of methods and analysis provided to allow replication by others?

Yes

Reviewer Expertise:

Cell biology, immunofluorescence microscopy, cancer biology, prostate cancer, bone metastasis, 3D cell culture models, inflammation, macrophages, gene expression, histology, vertical transmission of SARS-CoV-2, placenta biology, wound healing, intracellular trafficking, MMP14, cell migration

I confirm that I have read this submission and believe that I have an appropriate level of expertise to confirm that it is of an acceptable scientific standard, however I have significant reservations, as outlined above.

# Response

**Published:** 2024-10-30

**Authors:** Naomi McGovern

**Affiliations:** Pathology, University of Cambridge, Cambridge, England, UK

We thank the reviewers for their insightful comments.

**Summary of the research and overall impression:**

 The research article “ *Human placental cells are resistant to SARS-CoV-2 infection and replication*” is an interesting, well-designed, small study that adds to our understanding of why vertical transmission of SARS-CoV-2 does not occur frequently. The authors determined cellular ACE2 expression (primarily) on RNA and protein level within first trimester and term placental tissue and tested SARS-CoV-2 infection for various placental cell types in an *in vitro* infection assay. Their findings support the discourse and existing evidence as to why vertical transmission of SARS-CoV-2 is rare.

**Major concerns:**

 This is a well-designed study with a clear methodology. Unfortunately, the abstract does not do justice to the work done. Most strikingly, there is a disconnect between the methods section of the abstract (which only refers to the scRNAseq), the results section of the abstract (which only talks about the infection assay findings), and the respective section in the main body of the article. Redrafting the abstract to make sure it is reflective on the full breadth of the study is highly advised.

**Response:** We agree and have edited the abstract accordingly.

 For the placental ACE2 protein expression analysis via immunofluorescence microscopy, the data shown would be more compelling if the study used a positive and negative control, for example the HeLa+ACE2 and HeLa WT cell lines used in other parts of the study. At a minimum, the raw microscope files (rather than just the tiff image files) could be provided. We agree with the reviewer that the addition of these controls for the microscopy analyses would add additional value. However, we do not have that data. We have now added the raw microscope files to the Repository: https://doi.org/10.17863/CAM.112022. This is indicated in the Data Availability section of the manuscript.

 Considering the work performed and the findings made in this study, the discussion appears to be too brief, lacking to address important points, and thereby underselling the research. The following points should/could be covered to give the discussion more depth:

- While differences in RNA vs. protein expression are common, could this have any biological meaning for the observations made in this study? 
**Response:** 
  - We have expanded on this point in the Discussion to write ‘The differences observed between RNA and protein expression may be due to several factors, that we have not explored further. This includes regulatory factors that control the translation of mRNA and the small sample size used for our microscopy analyses.’
- What could be the cause of discrepancies in infection rate vs virion release in JEG3 vs primary trophoblasts (other than just calling them a “poor model”)?
**Response:** We agree that these observations were not fully explained. We have now added to the Discussion: ‘Both JEG-3 and JAR cells are carcinoma cell lines that historically, have been commonly used to study trophoblast biology due to the lack of alternative models. JEG-3 expresses HLA-G, while JAR is HLA class I negative. Based on this phenotype, they are widely used as models of primary extra villous trophoblast and villous trophoblast respectively.  However, their transcriptomes significantly differ to primary trophoblast cells ^1^. The findings from this study illustrate that the interaction of SARS-CoV-2 with JEG-3 and primary trophoblast cells differ. JAR cells, in contrast, demonstrate a similar level of resistance to infection as primary trophoblast cells. The mechanistic reasons for these observations have not been fully explored in this study and may be to several factors such as differences in anti-viral response genes or the fitness of cultured primary trophoblast cells. It is evident that results from cell mono-cultures, such as those used in this study, should be interpreted with caution. Furthermore, donor-to-donor variability may not have been fully captured with the small sample size of this study.’
- Can you also put the findings on infection rates of the other cell types investigated into the context of the literature?
**Response: **The infection rate of purified Hofbauer cells has not been previously investigated. Others have found that ACE2 expression by endothelial cells (and HUVECs) is low and accordingly these cells are resistant to infection by SARS-CoV-2 ^2–4^.  
  What implications (if any) does the use of the specific SARS-CoV-2 strain used in this study have on the findings? Would using a different strain change any of the findings? Why/why not?
**Response: **We thank the Reviewer for highlighting these Discussion points. We have added to the Discussion: ‘This study utilised the B.1.1.7 (Alpha) variant of concern. This lineage exhibits an enhanced rate of entry compared to the reference [Wuhan] strain of SARS-CoV-2 ^5^, although it is not as rapid as some of the variants of concern which subsequently arose (for example, B.1.617.2 (Delta)) ^6,7^. The isolate in question will undoubtably affect comparisons between studies. For example, there have been reports of increased rates of placentitis and stillbirth occurring during maternal infections with the B.1.617.2 (Delta) variant, compared to earlier variants ^8–11^ although it is important to note that the actual case numbers, as a percentage of pregnant infections, remain very low. It therefore remains possible that the use of a different isolate may have yielded different results.   Notably, infection by the B.1.617.2 (Delta) variant requires a lower threshold level of ACE2 on the host cell surface, leading to more efficient membrane fusion ^12^; it is therefore highly plausible that B.1.617.2 (Delta) may be able to enter some of the cell lines in this study which B.1.1.7 (Alpha) could not. This may also contribute to the altered observed disease spectrum, as it may allow B.1.617.2 (Delta) isolates to enter full term placental cells, whereas other variants may be limited to first trimester SCT which express higher ACE2 levels (Figure 1G).   The B.1.617.2 (Delta) variant is known to both enter cells (due to the presence of specific mutations in spike) and replicate the viral RNA more quickly in numerous cell models than early lineage strains ^7^. However, being able to enter a host cell rapidly does not necessarily translate to better overall transmission of the virus in question (and equally, it does not necessarily equate to worse disease outcomes; see for example the picornavirus coxsackievirus ^13^). A virus may be able to enter a cell but not uncoat or replicate efficiently, leading to low released virus titres and limited onwards transmission.   Conversely, a virus may enter ‘slowly’ and yet following RNA replication, packaging and budding it may traffic quickly to the plasma membrane and result in high virus titres. Elucidating the various contributions of these aspects is a complex task for any cell model.   This may explain the discrepancies we observed between virus entry and subsequent release in the JEG-3 cells, which were only mildly susceptible to infection and yet able to sustain efficient replication and egress (Figure 3B, C). They may express a range of pro-viral host factors at higher levels than the HeLa cells. Conversely, at every stage of the life cycle, a virus will encounter host antiviral restriction factors, targeting steps ranging from entry and uncoating ^14^ to egress ^15^. As many of these antiviral factors are the products of interferon stimulated genes ^16^, being upregulated rapidly during virus infections, the influence of host genetics will also contribute significantly to disease progression and will contribute to inter-study variations.   Related to this, it has been suggested that complications and poor neonatal outcomes arising from SARS-CoV-2 infections during pregnancy are a result of inflammatory responses in the placenta, rather than infection of either the neonate or placental tissue ^17,18^. The placental inflammatory responses to infection, or to circulating inflammatory cytokines, were not assessed in this study. Future studies should be targeted towards unravelling the effects of virus-induced inflammation upon placental cells, in addition to assessing virus entry by other variants.’  
- What are the limitations of the study? Future directions?
**Response: **The limitations of this study are mentioned throughout the discussion. Specially we write ‘ The differences observed between RNA and protein expression may be due to several factors, that we have not explored further. This includes regulatory factors that control the translation of mRNA and the small sample size used for our microscopy analyses. Furthermore, our transcriptomic data analyses did not take into consideration splice variants.’ We also discuss that other SARS-CoV-2 entry pathways that we have not analysed in this study may also be important. We discuss that only one variant of SARS-CoV-2 has been used in this study. A more complete study would have included other variants. We discuss the limitations of using JEG-3 and JAR cell lines and how other models such as trophoblast organoids would be of greater value to the field.   
**Minor suggestions:**
  Depending on the audience the authors hope to target, a paragraph on placental architecture, structures, and cell types would ensure that a wide range of readers have all relevant background information required to fully appreciate the study findings.  
**Response: **We agree and have added an additional paragraph explaining the placenta:   ‘The placenta is the first organ made by the fetus. It is a highly effective barrier, that restricts the vertical transmission of pathogens during pregnancy. Only a small selection of pathogens can bypass the defensive mechanisms of the placenta ^19^. Key to protecting the placenta from infection are the specific properties of the trophoblast. Trophoblast cells develop from the trophectoderm of the blastocyst and are the interface between the mother and fetus throughout pregnancy. Villous cytotrophoblast cells rapidly proliferate to form branching villi covered in multinucleated syncytiotrophoblast (SCT) layer that is the site of nutrient and oxygen exchange with maternal blood. Within the stroma of the villi, macrophages termed Hofbauer cells first appear at ~18 days post conception ^20^. These remain the major immune cells of the placenta throughout pregnancy.’  
  To further add to the data availability, it could be helpful to provide (exemplary) images of the CD31 / CK7 masks that were used to quantify ACE2 protein expression in the immunofluorescence of placental tissues.  
**Response **These data are include in the raw microscopy files in the Repository: https://doi.org/10.17863/CAM.112022
  Please indicate the type of error bar that is presented in Figure 2C in the figure legend.  
**Response: **Thanks for highlighting this omission. We have edited the Figure legend to ‘Mean +/- SEM’.
  In the results section “Placental cells are resistant to SARS-CoV-2 infection”, the specific reference to protein expression could be made to further strengthen the rationale for aspects of the infection assay: “ *For this assay, we focused on term placental trophoblast cells, due to their trend for higher levels of ACE2 [ **protein**] expression than first trimester trophoblast cells.*”  
**Response: **We agree and have edited the text to make it clearer.
  In Figure 3A, the figure legend indicates that Hoechst stain was used, but in the figure itself, it labels the blue channel as “DAPI”.  
**Response: **Hoechst stain was used. The Figure has been corrected.
  In Figure 3B and C, it might be better to remove the “WT” superscript from “HeLa(WT)+ACE2”.
**Response: ** We have removed the “WT” superscript.   

# Reviewer report: Reviewer response for version 1

**DOI:** 10.21956/wellcomeopenres.22708.r84519

**Published:** 2024-06-14

**Authors:** Connor Bamford

**Affiliations:** 1 Queen's University Belfast, Belfast, UK

**Review:**

 A focused in vitro study looking at in vitro infection and release with a very early isolate of SARS2 in reference to expression of two key entry factors, ACE2/TMPRSS2. They find that primary placental cells have reduced susceptibility to infection than controls likely due to expression less receptors. However, a commonly used placental cell line, JEG-3 can be infected and release infectious virus, unlike primary counterparts suggesting it is a poor model for placental infection biology.

**Abstract:**

 Abstract needs attention as not broken adequately into required sections (E.g. some methods not introduced in methods section and method section currently has results).

 Abstract and title should be clearer that this study is focused on in vitro experiments

**Introduction:**

 Increased risk factor for *reduced* maternal and foetal health?

 Define COVID-19

 What is rationale for only focusing on certain entry dependency factors and not considering other genes, especially known inhibitors of infection?

**Methods:**

 Is anything known about biological characteristics of placental donors, such as ethnicity or age?

**Results:**

 Have the gene expression results taken into consideration ACE2 splice variants?

**Discussion:**

 Be explicit about what you mean by SARS-CoV-2 has been found in placental tissue – is this RNA, infectious virus, infected cells, virions?

 Discuss the variability within individuals that is greater than other comparisons

 It would have been helpful to include a virus control for cell infectivity to know if these cultures are truly viable.

Is the work clearly and accurately presented and does it cite the current literature?

Yes

If applicable, is the statistical analysis and its interpretation appropriate?

Yes

Are all the source data underlying the results available to ensure full reproducibility?

Yes

Is the study design appropriate and is the work technically sound?

Partly

Are the conclusions drawn adequately supported by the results?

Yes

Are sufficient details of methods and analysis provided to allow replication by others?

Yes

Reviewer Expertise:

virology, infection, immunity

I confirm that I have read this submission and believe that I have an appropriate level of expertise to confirm that it is of an acceptable scientific standard.

# Response

**Published:** 2024-10-30

**Authors:** Naomi McGovern

**Affiliations:** Pathology, University of Cambridge, Cambridge, England, UK

We thank the reviewer for their insightful comments

**Review:**

 A focused in vitro study looking at in vitro infection and release with a very early isolate of SARS2 in reference to expression of two key entry factors, ACE2/TMPRSS2. They find that primary placental cells have reduced susceptibility to infection than controls likely due to expression less receptors. However, a commonly used placental cell line, JEG-3 can be infected and release infectious virus, unlike primary counterparts suggesting it is a poor model for placental infection biology.

**Abstract:**

 Abstract needs attention as not broken adequately into required sections (E.g. some methods not introduced in methods section and method section currently has results).

 Abstract and title should be clearer that this study is focused on in vitro experiments

**Response: **Thanks for highlighting this error. We have amended the Abstract as requested.

**Introduction:**

 Increased risk factor for *reduced* maternal and foetal health?

**Response: **This omission has been added.

 Define COVID-19

**Response: **We have added to the introduction “Coronavirus Disease 2019 (COVID-19) is a highly contagious respiratory illness caused by the severe acute respiratory syndrome coronavirus 2 (SARS-CoV-2)”

 What is rationale for only focusing on certain entry dependency factors and not considering other genes, especially known inhibitors of infection?

**Response: **In the Discussion we explain “Successful infection of a host cell depends on the presence of various surface host proteins, including but not limited to ACE2 and TMPRSS2. Following ACE2 engagement, S2’ site cleavage may be mediated by TMPRSS2 (allowing entry at the cell surface) or other proteases including cathepsin L, after endocytosis of the virion (reviewed in ^21^). Given the extremely low levels of TMPRSS2 expression which we observed (Figure 1H), it is possible that any entry in placental tissue is mediated by this latter pathway, although this study did not aim to elucidate which entry route was being utilised.”

**Methods:**

 Is anything known about biological characteristics of placental donors, such as ethnicity or age?

**Response: **We explain in the Methods that “Placentas were obtained from healthy women with apparently normal pregnancies undergoing elective first trimester terminations (6-12 weeks estimated gestational age (EGA) (n=20)). Term placentas were collected from healthy women with normal pregnancies and delivered by elective Cesarean section”. The ethnicity and age of the donors have not been recorded.

**Results:**

 Have the gene expression results taken into consideration ACE2 splice variants?

**Response: **No, the results have not taken into consideration splice variants. We have noted this is a limitation of the study in the Discussion.

**Discussion:**

 Be explicit about what you mean by SARS-CoV-2 has been found in placental tissue – is this RNA, infectious virus, infected cells, virions?

 Discuss the variability within individuals that is greater than other comparisons. We have added to the Discussion that the small sample size is a limitation of the study.

 It would have been helpful to include a virus control for cell infectivity to know if these cultures are truly viable.

**Response: **We used established culture protocols for primary placental cells ^22–24^. This been more clearly explained in the Method section.

 References:  

 1. Apps, R. *et al.* Genome-wide expression profile of first trimester villous and extravillous human trophoblast cells. *Placenta* 32, 33–43 (2011).

 2. Kumar, S. *et al.* Proteomic Analysis Identifies Molecular Players and Biological Processes Specific to SARS-CoV-2 Exposure in Endothelial Cells. (2022) doi:10.3390/ijms231810452.

 3. Schimmel, L. *et al.* Endothelial cells are not productively infected by SARS-CoV-2. *Clin Transl Immunology* (2021) doi:10.1002/cti2.1350.

 4. Agostinis, C. *et al.* SARS-CoV-2 modulates virus receptor expression in placenta and can induce trophoblast fusion, inflammation and endothelial permeability. (2022) doi:10.3389/fimmu.2022.957224.

 5. Dicken, S. J. *et al.* Characterisation of B.1.1.7 and Pangolin coronavirus spike provides insights on the evolutionary trajectory of SARS-CoV-2. *BioRxiv* (2021) doi:10.1101/2021.03.22.436468.

 6. Zhang, J. *et al.* Membrane fusion and immune evasion by the spike protein of SARS-CoV-2 Delta variant. *Science (1979)* 374, 1353–1360 (2021).

 7. Mlcochova, P. *et al.* SARS-CoV-2 B.1.617.2 Delta variant replication and immune evasion. *Nature* 599, 114–119 (2021).

 8.Shook, L. L. *et al.* SARS-CoV-2 Placentitis Associated With B.1.617.2 (Delta) Variant and Fetal Distress or Demise. *J Infect Dis* 225, 754–758 (2022).

 9. Knight, M. *et al.* Open access Original research Severity of maternal infection and perinatal outcomes during periods of SARS-CoV-2 wildtype, alpha, and delta variant dominance in the UK: prospective cohort study. 1, 53 (2022).

 10. DeSisto, C. L. *et al.* Risk for Stillbirth Among Women With and Without COVID-19 at Delivery Hospitalization — United States, March 2020–September 2021. *MMWR Morb Mortal Wkly Rep* 70, 1640–1645 (2021).

 11.  Shanes, E. D. *et al.* Placental Pathology After SARS-CoV-2 Infection in the Pre-Variant of Concern, Alpha / Gamma, Delta, or Omicron Eras. *Int J Surg Pathol* 31, 387–397 (2023). 12. Zhang, J. *et al.* Membrane fusion and immune evasion by the spike protein of SARS-CoV-2 Delta variant. *Science (1979)* 374, 1353–1360 (2021).

 13.  Lanahan, M. R., Maples, R. W. & Pfeiffer, J. K. Tradeoffs for a viral mutant with enhanced replication speed. *Proceedings of the National Academy of Sciences* 118, (2021).

 14. Shi, G. *et al.* Opposing activities of IFITM proteins in SARS‐CoV‐2 infection. *EMBO J* 40, (2021). 15.Stewart, H. *et al.* Tetherin antagonism by SARS‐CoV ‐2 ORF3a and spike protein enhances virus release. *EMBO Rep* 24, (2023).

 16.       Martin-Sancho, L. *et al.* Functional landscape of SARS-CoV-2 cellular restriction. *Mol Cell* 81, 2656-2668.e8 (2021).

 17. DeBiasi, R. L. Severe Acute Respiratory Syndrome-Coronavirus-2 Effects at the Maternal-Fetal Interface. *J Infect Dis* 225, 745–747 (2022).

 18. Guan, M. *et al.* Intrauterine Fetal Demise in the Third Trimester of Pregnancy Associated With Mild Infection With the SARS-CoV-2 Delta Variant Without Protection From Vaccination. *J Infect Dis* 225, 748–753 (2022).

 19. Megli, C. J. & Coyne, C. B. Infections at the maternal–fetal interface: an overview of pathogenesis and defence. *Nat Rev Microbiol* 20, 67–82 (2022). 20.        Boyd, J. D. & Hamilton, W. J. *The Human Placenta*. *Nursing mirror and midwives journal* vol. 134 (Nurs Mirror Midwives J, 1972).

 21. Jackson, C. B., Farzan, M., Chen, B. & Choe, H. Mechanisms of SARS-CoV-2 entry into cells. *Nat Rev Mol Cell Biol* 23, 3–20 (2022).

 22. Thomas, J. R. *et al.* Primitive haematopoiesis in the human placenta gives rise to macrophages with epigenetically silenced HLA-DR. *Nat Commun* 14, 1764 (2023).

 23.  Aye, I. L. M. H., Jansson, T. & Powell, T. L. Interleukin-1β inhibits insulin signaling and prevents insulin-stimulated system A amino acid transport in primary human trophoblasts. *Mol Cell Endocrinol* 381, 46–55 (2013).

 24.  Sheridan, M. A. *et al.* Characterization of primary models of human trophoblast. (2021) doi:10.1242/dev.199749 .
